# Factors associated with short sleep duration in adolescents

**DOI:** 10.1016/j.rppede.2015.10.007

**Published:** 2016

**Authors:** Érico Pereira Gomes Felden, Douglas Filipin, Diego Grasel Barbosa, Rubian Diego Andrade, Carolina Meyer, Fernando Mazilli Louzada

**Affiliations:** a Universidade do Estado de Santa Catarina (Udesc), Florianópolis, SC, Brazil; b Universidade Federal do Paraná (UFPR), Curitiba, PR, Brazil

**Keywords:** School health, Sleep, Habits, Adolescents

## Abstract

**Objective:**

This study aimed to investigate the prevalence and factors associated with short sleep duration in adolescents from Maravilha – Santa Catarina (SC), southern Brazil.

**Methods:**

The sample consisted of 516 adolescents aged 10–19 years of both genders. Issues associated with short sleep duration and difficulty falling asleep, chronotype, daytime sleepiness, physical activity, sedentary behavior and weight status were investigated.

**Results:**

The prevalence of short sleep duration (<8h on school days) was 53.6%. Adolescents aged 17–19 years showed a 2.05-fold (95%CI: 1.20–3.50) greater prevalence of short sleep duration than those aged 10–12 years. The ones studying in morning and evening shifts had a higher prevalence of short sleep duration compared to those in the afternoon shift. Older age and school shift were the main factors associated with short sleep duration.

**Conclusions:**

Adolescents from Maravilha showed high prevalence of short sleep duration, and older adolescents that studied in the morning and evening shifts showed reduced sleep.

## Introduction

Human beings go through transformations throughout life, both in physical aspect and in behavior.^1^ In this context, especially in adolescence, it is possible to observe significant changes in the expression of the sleep/wake cycle, including a delay in the sleep phase, characterized by going to sleep and waking up at late hours.^2^^,^ 3 This biological tendency may be exacerbated by behaviors such as the use of electronic media during the night, which, added to social engagements early in the morning, increase the prevalence of short sleep duration in this population.^4^

Over the years, in adolescence, the prevalence of short-duration, poor quality of sleep and excessive daytime sleepiness tends to progressively increase.^1^^–^ 8 This trend was identified in the study by Bernard et al.,^9^ with adolescents from São Paulo. The authors observed that the prevalence of short sleep duration was 5% at 10 years of age, 14% at 12 years, reaching 59% at the end of adolescence.

Recent studies have been trying to understand which factors are associated with the reduction of sleep hours in adolescence.^3^^,^ 8 ^,^ 10 ^–^ 12 In this context, some biological variables, as well as social and behavioral ones, are analyzed in order to better understand the expression of the sleep/wake cycle at this stage of life. This issue becomes more important as the short sleep duration, particularly during adolescence, is associated with cognitive deficits and decrease in health status.^13^^–^ 15

Considering the negative effects of short sleep duration in adolescents and the few population-based studies found in Brazil, especially in small municipalities, this study aimed to investigate the prevalence and factors associated with short sleep duration in adolescents from the municipality of Maravilha, in the state of Santa Catarina, southern Brazil.

## Method

The sample consisted of 516 adolescents (263 males), with a mean age of 14.57 (1.77) years. This sample was based on a population of 2969 adolescents aged 10–19 years, of both genders, regularly enrolled in public schools in the municipality of Maravilha, state of Santa Catarina, southern Brazil in 2013.^16^

The sample size was defined using the proposal of Luiz and Magnanini,^17^ considering a sampling error of five percentage points and 1.5 for the design effect. Based on this calculation, a minimum of 513 adolescents was estimated to constitute a representative school-based sample in the municipality. Sample selection was performed by clusters and proportional to the age groups of 10–14 and 15–19 years, taking into account the population of students in the final years of elementary school and in the three years of high school.

The adolescents answered a structured questionnaire containing questions on the following factors: sociodemographic, related to sleep and health.

As for sociodemographic factors, the following were assessed: gender, age, place of residence (rural or urban), school shift (morning, afternoon and evening), income (minimum wages received by the family) and schooling of the household head. Regarding income, the adolescents were classified as low income (up to 3 minimum wages – MW), middle income (3–6 MW) and high income (more than 6 MW).

Regarding factors related to sleep, the following data were analyzed: sleep duration was evaluated according to the time in bed, based on the times when going to sleep and waking up on school days. The adolescents with less than 8 h a day in bed were considered as having short sleep duration.^3^^,^ 9 ^,^ 18 Additionally, the time when going to sleep and waking up were investigated considering specific days of the week: from Monday to Thursday, from Friday to Saturday, from Saturday to Sunday, and from Sunday to Monday. The difficulty in falling asleep was investigated through the question “Do you have difficulty in falling asleep?”, with the following response options: (a) never, (b) sometimes, (c) always.^3^

Daytime sleepiness was assessed using the Pediatric Daytime Sleepiness Scale (PDSS).^19^ This scale consists of eight multiple-choice questions. Each question has five response options, using a Likert scale: 0=never; 1=almost never; 2=sometimes; 3=frequently and 4=always. At the end, question scores were summed, with the scale score ranging from zero to 32. High scores indicate more daytime sleepiness. As there are no classifications for this scale, the adolescents were allocated considering the tertiles. Thus, those adolescents at the third tertile (highest score of PDSS) were classified as having more daytime sleepiness.

Chronotype was investigated using the Munich Chronotype Questionnaire (MCTQ).^20^ In this questionnaire, the chronotype definition is given as a phase of the sleep/wake cycle, represented by the corrected half-sleep phase, also considering the free days. The chronotype in MCTQ is given in hours, ranging from 0 to 12 h; lower values represent the morningness, while higher values represent eveningness. The adolescents were divided into tertiles considering the MCTQ score, with the first tertile representing those with morning behavior.

Finally, regarding health-related factors, the perception of health and stress, weight status, physical activity level and time in sedentary behaviors were investigated. Sedentary behavior was inferred by the time spent in the sitting position, which was investigated by the question “How long do you usually spend in the sitting position on a weekday?”.^21^ This question was extracted from the “International Physical Activity Questionnaire” and considers in its analysis the time during which the adolescents remain seated during the day, at work, at school or college, at home and during their free time, including the time spent studying, resting, doing homework, visiting a friend, reading, sitting or lying down while watching TV. This analysis does not include the time spent sitting during transport by bus, train, subway or car. As in the analysis of sleepiness and chronotype, the specialized literature does not have a specific cutoff for the long or short time of sedentary behavior. Therefore, the strategy of using tertiles was also adopted for this variable.

The subjective perception of adolescents’ health was investigated through the question: “How do you classify your current health status?”. For this question, a positive health perception was considered for the answers “excellent” and “good”, and a negative health perception for the answers “regular” and “bad”.^22^

This same strategy was adopted to investigate the perception of stress, which was inferred by the question: “How would you describe the level of stress in your life?”. The following response options were considered: rarely stressed (living very well); sometimes stressed (living fairly well); often stressed (often facing problems) and excessively stressed (difficulty to cope with daily life). Adolescents that answered the question with the alternatives “often stressed” and “excessively stressed” were considered as having a high-stress perception.

As for the weight status variable, height was measured using a stadiometer, fixed vertically on the wall, and the body mass was measured using a digital scale with 100 g of precision, following the recommended measurement procedures by Alvarez and Pavan.^23^ The body mass index (BMI) was obtained from the ratio between body mass (kg) and squared height (m). The categorization of this variable was carried out considering the criteria proposed by the International Obesity Task Force, by age and gender,^24^ complemented by scores indicative of low weight in Cole et al.^24^^,^ 25 Weight status was categorized as underweight, normal weight, overweight and obesity.

The level of physical activity was investigated through the questionnaire proposed by Florindo et al.^26^ This questionnaire consists of 17 questions on habitual physical activity, considering the total time in minutes and weekly, and was validated considering the cardiorespiratory fitness. The adolescents were classified as insufficiently active if they performed less than 300 min of weekly physical activity.^27^

Descriptive analyses were performed (means, frequency and standard deviations). The Kolmogorov–Smirnov test indicated that the analyzed data were nonparametric. Thus, the Kruskal–Wallis test was used to compare continuous variables, whereas the Chi-square test was used to verify the association between proportions. Due to the high prevalence observed of short sleep duration, Poisson regression was carried out to investigate associations between the variables.^28^ The adjusted model considered the variables gender, age range, school shift, family income, daytime sleepiness, naps and time spent in the sitting position with *p*<0.25 in the crude analysis.^29^ Considering this analysis, the following variables were excluded from the adjusted model: place of residence, chronotype and physical activity. A significance level of 5% was considered for all analyses.

The Informed Consent form was given to all adolescents younger than 18 to be signed by parents/guardians, and all adolescents involved in the study received the Informed Assent form. The project was evaluated by the institution's Institutional Review Board, according to Edict N. 535,621/2013.

## Results

The sample descriptive data are shown in Table 1. The investigated adolescents were, on average, 14.6±1.7 years old and most of them lived in the urban area of the municipality. The assessed adolescents remained a mean of 382.2 min a day in the sitting position, and the female adolescents spent more time than males in this behavior (*p*=0.001). The girls had lower levels of physical activity (*p*=0.003) and a higher prevalence of excessive stress perception (*p*<0.001) when compared to boys.

**Table 1:** Descriptive data of the sample with differences between genders.

Variable		Total	Male	Female	*p* -value a
Age (years)		14.6 (1.8)	14.4 (1.9)	14.7 (1.6)	0.033
*Age range (%)*					0.041
	10–12	15.3	18.6	11.9	
	13–14	27.3	29.7	24.9	
	15–16	45.5	40.3	51.0	
	17–19	11.8	11.4	12.3	

*Place of residence (%)*					0.662
	Rural	21.2	20.9	19.4	
	Urban	79.8	79.1	80.6	

*School shift (%)*					0.782
	Morning	71.9	71.1	72.7	
	Afternoon	19.4	20.5	18.2	
	Evening	8.7	8.4	9.1	

*Family income (%)*					0.642
	≤3 minimum wages	20.0	23.2	16.9	
	3.1–6 minimum wages	50.2	45.6	54.9	
	>6 minimum wages	29.8	31.2	28.5	

*PDSS (points)*		15.1 (5.6)	14.6 (5.4)	15.6 (5.7)	0.030
*Munich* b *(hours)*		4.6 (1.67)	4.7 (1.75)	4.4 (1.58)	0.028

*Nap (%)*					0.084
	Never/almost never	33.5	36.9	30.0	
	Sometimes	57.6	52.9	62.5	
	Almost always/always	8.9	10.3	7.5	

*Weekly physical activity (min.)*		501.5 (536.6)	562.9 (523.9)	437.6 (543.3)	<0.001
*Insufficiently active (%)*		40.1	33.5	46.2	0.003
*Time spent in the sitting position (min.)*		382.2 (234.1)	352.1 (225.8)	413.6 (238.9)	0.001
*Perception of high stress*		22.1	11.8	32.8	<0.001

*Weight status*					0.493
	Normal weight	81.0	79.8	82.2	
	Excess weight	19.0	20.2	17.8	

a *p* -value of Chi-square or Kruskal–Wallis.

b Chronotype according to Munich's classification.

PDSS, Pediatric Daytime Sleepiness Scale.

**Figure 1:**
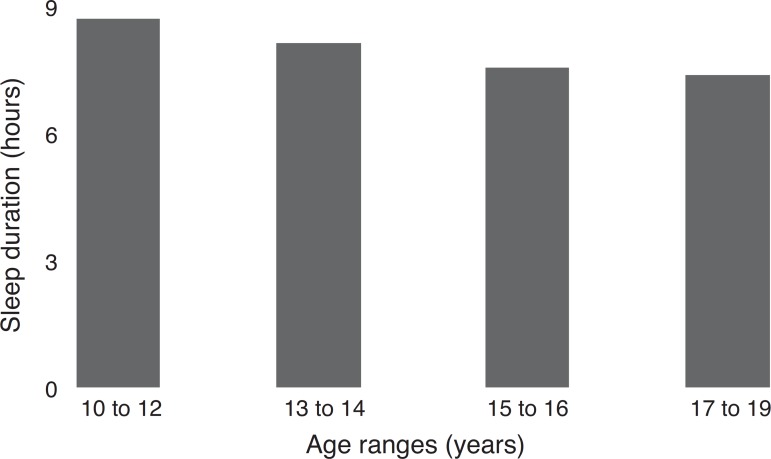
Sleep duration decrease with increasing age.

**Figure 2:**
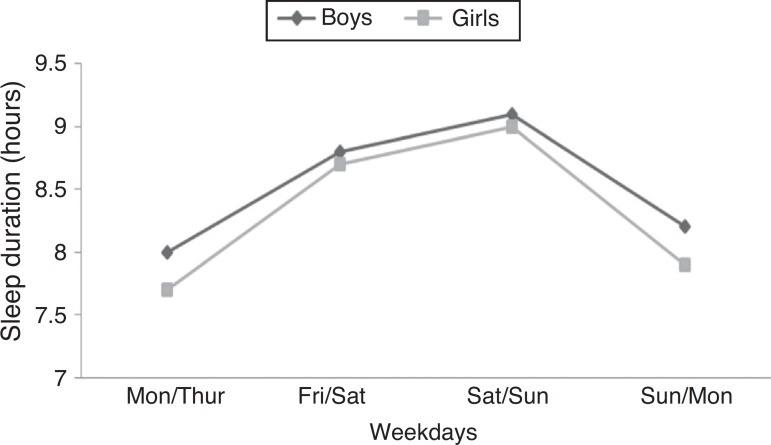
Increased sleep duration on weekends.

Mean sleep duration was 7.9±1.6 h and no differences were identified between genders (*p*=0.216). The prevalence of short sleep duration (less than 8 h) was 53.6%, with differences between age groups (*p*<0.001). Sleep duration during adolescence and on different days of the week is shown in Figs. 1 and 2.

Fig. 2 shows a trend of decreasing hours of sleep throughout adolescence. While the prevalence of short sleep duration at 10–12 years was 31.2%, in the age group of 17–19 years the prevalence increased to 70%. Additionally, as shown in Fig. 2, the adolescents tended to have longer sleep duration during the weekend.

Table 2 shows the association analysis between short sleep duration and the independent variables. The adjusted analysis showed that the age range variable was strongly associated with the outcome. Therefore, adolescents aged 15–16 and 17–19 had respectively; 1.71- (95%CI: 1.09–2.69) and 2.05-fold (95%CI: 1.20–3.50) higher prevalence of short sleep duration, when compared to the age group of 10–12 years.

**Table 2:** Poisson regression analysis considering short sleep duration as the dependent variable.

Variable		Prevalence (%)	Unadjusted analysis	Adjusted analysis
			PR (95%CI)	PR (95%CI)
*Gender*				
	Male	51.0	1	1
	Female	56.4	1.10 (0.94–1.30)	1.02 (0.80–1.32)

*Age range*				
	10–12	31.2	1	1
	13–14	46.8	1.50 (1.03–2.18)	1.46 (0.91–2.34)
	15–16	60.9	1.95 (1.38–2.76)	1.71 (1.09–2.69)
	17–19	70.0	2.24 (1.55–3.25)	2.05 (1.20–3.50)

*Place of residence*				Excluded
	Rural	48.5	1	
	Urban	54.9	1.13 (0.91–1.41)	

	*School shift*			
	Morning	58.3	1.82 (1.35–2.46)	1.59 (0.93–2.71)
	Afternoon	32.0	1	1
	Night	63.6	1.98 (1.38–2.86)	1.68 (1.14–2.46)

*Family income*				
	≤3 minimum wages	49.3	1	1
	3.1–6 minimum wages	51.9	1.05 (0.85–1.29)	1.03 (0.76–1.39)
	>6 minimum wages	60.9	1.23 (1.00–1.53)	1.19 (0.86–1.64)

*PDSS*				
	1st tertile	47.1	1	1
	2nd tertile	54.7	1.16 (0.95–1.43)	1.05 (0.77–1.41)
	3rd tertile	59.7	1.26 (1.03–1.56)	1.10 (0.79–1.52)

	*Chronotype*			Excluded
	1st tertile	51.9	1	
	2nd tertile	50.3	0.97 (0.78–1.19)	
	3rd tertile	57.7	1.14 (0.91–1.36)	

*Nap*				
	Never/almost never	44.8	1	1
	Sometimes	56.7	1.26 (1.04–1.54)	1.13 (0.86–1.49)
	Almost always/always	67.4	1.50 (1.16–1.95)	1.23 (0.79–1.92)

*Physical activity*				Excluded
	Active	51.9	1	
	Insufficiently active	56.2	1.08 (0.92–1.27)	

*Time spent in the sitting position*				
	1st tertile	47.3	1	1
	2nd tertile	57.1	1.20 (1.00–1.47)	1.01 (0.74–1.38)
	3rd tertile	61.2	1.29 (1.07–1.57)	1.09 (0.80–1.49)

PDSS, Pediatric Daytime Sleepiness Scale.

Another factor strongly associated with short sleep duration was the school shift. Adolescents who studied in the morning shift had 1.82-fold (95%CI: 1.35–2.46) higher prevalence of short sleep duration, when compared to students in the afternoon shift. In the adjusted analysis, adolescents who studied in the evening shift had 1.68-fold (95%CI: 1.14–2.46) higher prevalence of short sleep duration in relation to adolescents who studied in the afternoon shift.

Although daytime sleepiness, napping and time in the sitting position did not remain associated with the outcome in the adjusted analysis, they showed significant associations with short sleep duration. Adolescents at the third tertile of the sleepiness scale had 1.26-fold (95%CI: 1.03–1.56) higher prevalence of short sleep duration than the ones at the first tertile. Napping was associated with lower sleep duration in the “sometimes” category (PR=1.26; 95%CI: 1.04–1.54) and in the “always” category (PR=1.50; 95%CI: 1.16–1.95), indicating that adolescents with shorter nocturnal sleep more frequently napped. The time spent in the sitting position showed associations in the second tertile (OR=1.20; 95%CI: 1.00–1.47) and in the third tertile (OR=1.29; 95%CI: 1.07–1.57), indicating that adolescents who spent more time in sedentary behaviors had shorter duration of sleep at night.

## Discussion

This study showed evidence of associations between short sleep duration and the adolescents’ school shift and age range. The prevalence of short sleep duration was of 53.6% among the assessed adolescents. This prevalence is high compared to other studies.^3^^,^ 9 ^,^ 12 ^,^ 30 Although the prevalence was high, higher percentages were observed in North-American adolescents.^11^ It is worth mentioning that the municipality of Maravilha – SC is small and has a high percentage of rural population, and this prevalence was not expected.

Adolescents with short sleep duration show an increase in several health risks. Javaheri et al.^31^ warn that short sleep duration and the poor quality of sleep are, in many cases, associated with increased presence of pathologies. Pereira et al.^10^ observed an association between short sleep duration and increased presence of stress in adolescents. Furthermore, as seen in the study by Nova et al.,^12^ Spanish adolescents with adequate sleep duration had a lower incidence of allergies.

There was a trend of increased prevalence of short sleep duration throughout adolescence. As described in the literature, age is strongly associated with short sleep duration, considering the maturation of the central nervous system and risk behaviors, such as using electronic media, especially at night.^10^^,^ 32 In adolescence, individuals are more likely to have a biological phenomenon called delayed sleep-phase, in which adolescents tend to sleep and wake up at late hours,^33^ which may contribute to increased daytime sleepiness, if sleep is not recovered during the day.^32^ Moreover, this biological tendency is exacerbated by certain behaviors which, added to early morning school commitments, markedly increase the number of adolescents with short sleep duration.^4^

Due to the aforementioned reasons, it can be emphasized that the adolescents attending daytime and nighttime school shifts showed a higher association with short sleep duration. Older adolescents and those studying at these shifts constitute a major risk group for short sleep duration. In the study by McKnight-Eily et al.,^11^ the authors propose, to diminish the high prevalence of short sleep duration, individual observation of adolescents, indicating change in behavior and even the change of the school shift. In this context, Louzada and Menna-Barreto^34^ discuss the possibility of starting classes at a later hour in the morning as a possible intervention. However, the same authors highlight the difficulty for schools to adopt these recommendations, considering the working hours of parents and even the culture of valuing activities that start early in the morning.

It was observed that the highest frequency napping was associated with short sleep duration in adolescents. Probably, adolescents who more frequently nap use this habit as a way to compensate for the short duration of nocturnal sleep. This result corroborates the findings of Bernard et al.,^9^ who also found a higher prevalence of short sleep duration in adolescents with a higher frequency of napping. Although this habit represents a way to compensate for sleep deficit, according to Carskadon et al.,^2^ it can delay the propensity to nighttime sleep, delaying its onset and reducing its duration. Therefore, it is necessary to investigate napping dose-response, considering its frequency and duration, for possible recommendations to adolescents.

Finally, it is noteworthy that adolescents with shorter sleep duration had higher time of sedentary behavior. Thus, as the literature indicates an increasing prevalence of short sleep duration, it is also possible to identify that adolescents spend more time in sedentary behaviors, especially in front of the TV and at the internet.^35^^,^ 36 It is therefore necessary to investigate whether those adolescents with shorter sleep duration prefer to spend more time in activities with low energy expenditure due to, for instance, excessive daytime sleepiness.

Positives points of this study can be highlighted, such as, the use of a representative sample of a small town in the countryside, rarely studied, as well as the discussion of associations not available in the national literature. The main limitation of this study is the use of a questionnaire to indirectly assess the issues of sleep and physical activity, which even though are validated tools, may lead to underestimated or overestimated measures by the adolescents.

The adolescents from Maravilha – SC showed a high prevalence of short sleep duration, and the older adolescents and the ones who studied in the morning and evening school shifts showed reduced sleep duration. This result indicates the need for actions aimed at schoolchildren's health focused on biological and behavioral profiles at different stages of adolescence. In this respect, it is worth mentioning that the adolescent may not notice changes in their sleep patterns, and thus educational measures are important since the start of the elementary school, helping them to establish and maintain adequate sleep hygiene. In this sense, it is recommended that classes should be offered in the afternoon for all school years and even the creation of an intermediate shift starting at midmorning.

Even though age and school shift were the factors most strongly associated with short sleep duration, the habit of napping, sedentary behaviors and daytime sleepiness are important variables that must be considered in the short sleep duration analysis. Thus, it is recommended to perform studies that will provide knowledge on the causal associations between these variables and to indicate, with higher precision, the adequate napping time, so that the adolescent will not have loss of nocturnal sleep. It is also essential to propose ways to minimize the effect of sleep deprivation on increased daytime sleepiness and time spent in sedentary behaviors, considering the adolescent's reality.
