# Supplementary material for: Factors associated with short sleep duration in adolescents
Source: Rev Paul Pediatr. 2016 Jan-Mar;34(1):64–70. doi: 10.1016/j.rppede.2015.10.007 (PMC4795723; doi:10.1016/j.rppede.2015.10.007)
Supplement: Translation

# Translation: Fatores associados à baixa duração do sono em adolescentes

**Authors:** Érico Pereira Gomes Felden, Douglas Filipin, Diego Grasel Barbosa, Rubian Diego Andrade, Carolina Meyer, Fernando Mazilli Louzada

**Affiliations:** a Universidade do Estado de Santa Catarina (Udesc), Florianópolis, SC, Brasil; b Universidade Federal do Paraná (UFPR), Curitiba, PR, Brasil

**Keywords:** Saúde escolar, Sono, Hábitos, Adolescentes

## Abstract

**Objetivo:**

Investigar a prevalência e os fatores associados à baixa duração do sono de adolescentes do município de Maravilha (SC), Brasil.

**Métodos:**

A amostra foi formada por 516 adolescentes, de 10 a 19 anos, de ambos os sexos. Foram investigadas questões associadas à baixa duração do sono, como dificuldade de pegar no sono, cronotipo, sonolência diurna, atividade física, comportamento sedentário e *status* de peso.

**Resultados:**

A prevalência de baixa duração do sono (<8h nos dias com aula) foi de 53,6%. Os adolescentes com 17-19 anos tiveram 2,05 (IC95% 1,20-3,50) vezes mais chances de apresentar baixa duração do sono do que os com 10-12 anos. Aqueles que estudavam nos turnos manhã e noite apresentaram maior prevalência de baixa duração de sono, com relação aos do turno da tarde. Idade mais avançada e turno escolar foram os principais fatores associados à baixa duração do sono.

**Conclusões:**

Os adolescentes de Maravilha apresentaram alta prevalência de baixa duração do sono. Os mais velhos, que estudam de manhã e à noite, apresentaram sono reduzido.

## Introdução

Ao longo da vida, o ser humano passa por transformações, tanto em sua forma física quanto em seu comportamento.^1^ Nesse contexto, em especial na adolescência, é possível observar importantes mudanças na expressão do ciclo vigília/sono que incluem um atraso na fase de sono, caracterizado por horários de dormir e acordar mais tardios.^2^^,^ 3 Essa tendência biológica pode ser exacerbada por comportamentos como o uso de mídias eletrônicas durante a noite, que, somado aos compromissos sociais no início da manhã, aumenta a prevalência de baixa duração do sono nessa população.^4^

Com o passar dos anos, na adolescência, as prevalências de baixa duração, má qualidade do sono e sonolência diurna excessiva tendem a aumentar progressivamente.^1^^-^ 8 No estudo de Bernardo et al.,^9^ com adolescentes paulistas, identificou-se essa tendência. Os autores observaram que, aos 10 anos, a prevalência de baixa duração do sono era de 5%, aos 12 anos de 14% e no fim da adolescência de 59%.

Pesquisas recentes tentam compreender quais fatores estão associados com a diminuição das horas de sono na adolescência.^3^^,^ 8 ^,^ 10 ^-^ 12 Nesse contexto, algumas variáveis, tanto de cunho biológico como social e comportamental, são analisadas com o objetivo de compreender melhor a expressão do ciclo vigília/sono nessa fase de vida. Essa temática ganha importância na medida em que a baixa duração do sono, especialmente na adolescência, está associada com os déficits cognitivos e com a diminuição da saúde.^13^^-^ 15

Tendo em vista os efeitos negativos da baixa duração do sono em adolescentes e devido aos poucos estudos de base populacional encontrados no Brasil, especialmente em municípios de pequeno porte, o presente estudo teve como objetivo investigar a prevalência e os fatores associados à baixa duração do sono em adolescentes do município de Maravilha do Estado de Santa Catarina.

## Método

Participaram da amostra 516 adolescentes (263 do sexo masculino), com idade média de 14,57 (1,77) anos. Essa amostra foi baseada em população de 2.969 adolescentes de 10 a 19 anos, de ambos os sexos, devidamente matriculados em 2013 em escolas públicas do município de Maravilha, SC, Brasil.^16^

O tamanho da amostra foi definido com base na proposta de Luiz e Magnanini,^17^ que considera um erro amostral de cinco pontos percentuais e efeito de delineamento de 1,5. A partir desse cálculo foi identificado o mínimo de 513 adolescentes para compor uma amostra representativa, de base escolar, do município. A seleção da amostra foi por conglomerado e proporcional às faixas de 10-14 e de 15-19 anos e levou em consideração a população de estudantes nas séries finais do ensino fundamental e dos três anos do ensino médio.

Os adolescentes responderam a um questionário estruturado com questões sobre os seguintes fatores: sociodemográficos, ligados ao sono e à saúde.

Quanto aos fatores sociodemográficos, foram investigados: sexo, idade, local de domicílio (rural ou urbano), turno de estudo (manhã, tarde e noite), renda (salários mínimos recebidos pela família) e grau de instrução do chefe de família (anos de estudo). Com relação à renda os adolescentes foram classificados em baixa renda (até três salários), média (entre três-seis salários) e alta renda (mais do que seis salários).

No que concerne aos fatores relacionados ao sono, os seguintes dados foram analisados: a duração do sono foi avaliada de acordo com o tempo na cama, a partir dos horários de dormir e acordar nos dias com aula. Foram considerados com baixa duração de sono os adolescentes que apresentaram tempo na cama menor do que oito horas por dia.^3^^,^ 9 ^,^ 18 Além disso, foram investigados os horários de dormir e acordar e consideraram-se dias específicos da semana: de segunda a quinta-feira, de sexta-feira para sábado, de sábado para domingo e de domingo para segunda-feira. A dificuldade para pegar no sono foi investigada por meio da pergunta “Você sente dificuldades para pegar no sono?”. O adolescente tinha três opções de resposta: a) nunca, b) às vezes, c) sempre.^3^

A sonolência diurna foi analisada por meio da Pediatric Daytime Sleepiness Scale (PDSS).^19^ É composta por oito questões de múltipla escolha. Cada questão tem cinco opções de resposta, com uma escala *likert*: 0=nunca; 1=quase nunca; 2=às vezes; 3=frequentemente e 4=sempre. No fim foi feito o somatório dos escores das questões e a pontuação da escala podia variar de 0 a 32. Pontuações mais altas indicam mais sonolência diurna. Como não existem classificações para essa escala, os adolescentes foram alocados considerando os tercis. Assim, aqueles adolescentes do terceiro tercil (maior pontuação da PDSS) foram classificados com mais sonolência diurna.

O cronotipo foi investigado a partir do Questionário de Munique (MCTQ).^20^ Nesse questionário a definição do cronotipo é dada como uma fase do ciclo vigília/sono, representada pela meia fase do sono corrigida e que considera também os dias livres. O cronotipo no MCTQ é dado em horas, varia de 0-12 horas; valores menores representam a matutinidade, enquanto os maiores representam a vespertinidade. Os adolescentes foram alocados em tercis que consideraram a pontuação do MCTQ. Os do primeiro tercil foram aqueles com comportamentos mais matutinos.

Finalmente, em relação aos fatores relacionados à saúde, foram investigados a percepção de saúde e de estresse, o *status* de peso, o nível de atividade física e o tempo em comportamentos sedentários. O comportamento sedentário foi inferido pelo tempo sentado, o qual foi investigado por meio da pergunta “Quanto tempo no total você gasta sentado durante um dia de semana?”.^21^ Essa pergunta foi extraída do Questionário Internacional de Atividade Física e considera em sua análise o tempo em que o adolescente permanece sentado durante o dia, no trabalho, na escola ou faculdade, em casa e durante seu tempo livre, incluindo o tempo sentado para estudar, enquanto descansa, fazer lição de casa, visitar um amigo, ler, sentado ou deitado, assistir a TV. Essa análise não inclui o tempo gasto sentando durante o transporte em ônibus, trem, metrô ou carro. Assim, como na análise da sonolência e do cronotipo, não existe na literatura especializada um ponto de corte específico para tempo de comportamento sedentário alto ou baixo. Dessa forma, foi adotada a estratégia de divisão em tercis que considerou também essa variável.

A percepção subjetiva de saúde dos adolescentes foi investigada por meio da seguinte pergunta: “Como você classifica seu estado de saúde atual?”. Para essa questão foram consideradas percepção positiva de saúde as respostas “excelente” e “boa” e percepção negativa de saúde as respostas “regular” e “ruim”.^22^

Essa mesma estratégia foi adotada para investigar a percepção de estresse, a qual foi inferida por meio da questão: “Como você descreve o nível de estresse em sua vida?”. Foram consideradas as seguintes opções de resposta: raramente estressado (vive muito bem); às vezes estressado (vive razoavelmente bem); quase sempre estressado (enfrenta problemas com frequência) e excessivamente estressado (com dificuldade para enfrentar a vida diária). Foram considerados adolescentes com percepção de alto estresse aqueles que responderam à pergunta com as opções “quase sempre estressado” e “excessivamente estressado”.

Para compor a variável *status* de peso, foram aferidas a estatura com estadiômetro fixado verticalmente na parede e a massa corporal com balança digital com escalas de resolução de 100 g, seguindo os procedimentos para medidas recomendados por Alvarez e Pavan.^23^ O índice de massa corporal (IMC) foi obtido a partir da razão entre a massa corporal (Kg) e a estatura (m) ao quadrado. A categorização dessa variável foi feita considerando os critérios propostos pela International Obesity Task Force, por idade e sexo,^24^ complementada pelos escores indicativos de baixo peso em Cole et al. 24 ^,^ 25 O *status* de peso foi categorizado em baixo peso, peso normal (eutrofia), sobrepeso e obesidade.

O nível de atividade física foi investigado por meio do questionário proposto por Florindo et al.^26^ É composto por 17 questões sobre atividades físicas habituais, considerado o tempo total em minutos e semanal, e foi validado com base na capacidade cardiorrespiratória. Os adolescentes foram classificados como insuficientemente ativos se fizessem menos de 300 minutos de atividades físicas semanais.^27^

Foram feitas análises descritivas (médias, frequências e desvios padrões). O teste de Komogorov-Smirnov indicou que os dados analisados não eram paramétricos. Assim, para comparar as variáveis contínuas, usou-se o teste de Kruskal-Wallis e para testar a associação entre as proporções o teste do qui-quadrado. Em função das altas prevalências observadas de baixa duração de sono, foi feita regressão de Poisson para investigar as associações entre as variáveis.^28^ No modelo ajustado, foram consideradas as variáveis sexo, faixa etária, turno de estudo, renda familiar, sonolência diurna, sesta e tempo sentado, que apresentaram *p*<0,25 na análise bruta.^29^ Considerando essa análise, foram excluídas do modelo ajustado as variáveis local de domicílio, cronotipo e atividade física. Para todas as análises considerou-se um nível significância de 5%.

Aos menores de 18 anos foi entregue o Termo de Consentimento Livre e Esclarecido para assinatura dos pais ou responsáveis e a todos os envolvidos (adolescentes) entregue o Termo de Assentimento Livre e Esclarecido. O projeto foi avaliado pelo comitê de ética envolvendo seres humanos da instituição de origem, conforme parecer n° 535.621/2013.

## Resultados

Os dados descritivos da amostra foram apresentados na tabela 1. Os adolescentes investigados apresentaram, em média; 14,6±1,7 anos e residiam, em sua maioria, na zona urbana do município. Os adolescentes pesquisados permaneciam, em média, 382,2 minutos sentados por dia. As adolescentes permaneciam mais tempo nesse comportamento (*p*=0,001). As moças tinham menores níveis de atividade física (*p*=0,003) e maior prevalência de percepção de alto estresse (*p*<0,001) quando comparadas com os rapazes.

**Tabela 1:** Dados descritivos da amostra com a diferença entre os sexos

Variável		Total	Masculino	Feminino	*p* ‐valor a
*Idade (anos)*		14,6(1,8)	14,4(1,9)	14,7(1,6)	0,033
*Faixas etárias (%)*					0,041
	10‐12	15,3	18,6	11,9	
	13‐14	27,3	29,7	24,9	
	15‐16	45,5	40,3	51,0	
	17‐19	11,8	11,4	12,3	
*Local de domicílio (%)*					0,662
	Rural	21,2	20,9	19,4	
	Urbano	79,8	79,1	80,6	
*Turno de estudo (%)*					0,782
	Manhã	71,9	71,1	72,7	
	Tarde	19,4	20,5	18,2	
	Noite	8,7	8,4	9,1	
*Renda familiar (%)*					0,642
	≤3 salários mínimos	20,0	23,2	16,9	
	3,1‐6 salários mínimos	50,2	45,6	54,9	
	>6 salários mínimos	29,8	31,2	28,5	
	*PDSS (pontos)*	15,1 (5,6)	14,6 (5,4)	15,6 (5,7)	0,030
	*Munick* b *(horas)*	4,6 (1,67)	4,7 (1,75)	4,4 (1,58)	0,028
*Sesta (%)*					0,084
	Nunca/quase nunca	33,5	36,9	30,0	
	Às vezes	57,6	52,9	62,5	
	Quase sempre/sempre	8,9	10,3	7,5	
	*Atividade física semana (min.)*	501,5 (536,6)	562,9 (523,9)	437,6 (543,3)	<0,001
	*Insuficientemente ativos (%)*	40,1	33,5	46,2	0,003
	*Tempo sentado (min.)*	382,2 (234,1)	352,1 (225,8)	413,6 (238,9)	0,001
	*Percepção de estresse alto (%)*	22,1	11,8	32,8	<0,001
Status *de peso*					0,493
	Eutróficos	81,0	79,8	82,2	
	Excesso de peso	19,0	20,2	17,8	

a p‐valor do qui‐quadro ou Kruskal‐Wallis.

b Cronotipo segundo classificação de Munick.

PDSS, Pediatric Daytime Sleepiness Scale.

A duração média do sono foi de 7,9±1,6 horas. Não foram identificadas diferenças nos sexos (*p*=0,216). A prevalência de baixa duração de sono (menos de oito horas) foi de 53,6%, com diferenças entre as faixas etárias (*p*<0,001). A duração de sono no decorrer da adolescência e nos diferentes dias da semana foi ilustrada nas figuras 1 e 2.

**Figura 1:** Diminuição da duração de sono com o aumento da idade.

**Figura 2:** Aumento da duração do sono nos fins de semana.

Na figura 2 é possível observar tendência de diminuição das horas de sono no decorrer da adolescência. Enquanto a prevalência de baixa duração do sono aos 10-12 anos foi de 31,2%, na faixa de 17-19 anos passou para 70%. Além disso, conforme a figura 2, os adolescentes tenderam a apresentar maior duração do sono no fim de semana.

Na tabela 2 encontra-se a análise de associação entre a baixa duração do sono e as variáveis independentes. Observou-se, na análise ajustada, que a variável faixa etária esteve fortemente associada ao desfecho. Dessa forma, os adolescentes da faixa de 15-16 e 17-19 apresentaram, respectivamente, 1,71 (IC95% 1,09-2,69) e 2,05 (IC95% 1,20-3,50) vezes maior prevalência de baixa duração de sono, em relação à faixa de 10-12 anos.

**Tabela 2:** Análise de regressão de Poisson que considera a baixa duração do sono como variável dependente

Variáveis		Prevalências (%)	Análise não ajustada	Análise ajustada
			RP (IC95%)	RP (IC95%)
*Sexo*				
	Masculino	51,0	1	1
	Feminino	56,4	1,10 (0,94‐1,30)	1,02 (0,80‐1,32)

*Faixas etárias*				
	10‐12	31,2	1	1
	13‐14	46,8	1,50 (1,03‐2,18)	1,46 (0,91‐2,34)
	15‐16	60,9	1,95 (1,38‐2,76)	1,71 (1,09‐2,69)
	17‐19	70,0	2,24 (1,55‐3,25)	2,05 (1,20‐3,50)

	*Local de domicílio*			Excluído
	Rural	48,5	1	
	Urbano	54,9	1,13 (0,91‐1,41)	

*Turno de estudo*				
	Manhã	58,3	1,82 (1,35‐2,46)	1,59 (0,93‐2,71)
	Tarde	32,0	1	1
	Noite	63,6	1,98 (1,38‐2,86)	1,68 (1,14‐2,46)

*Renda familiar*				
	≤3 salários mínimos	49,3	1	1
	3,1‐6 salários mínimos	51,9	1,05 (0,85‐1,29)	1,03 (0,76‐1,39)
	>6 salários mínimos	60,9	1,23 (1,00‐1,53)	1,19 (0,86‐1,64)

*PDSS*				
	1° tercil	47,1	1	1
	2° tercil	54,7	1,16 (0,95‐1,43)	1,05 (0,77‐1,41)
	3° tercil	59,7	1,26 (1,03‐1,56)	1,10 (0,79‐1,52)

	*Cronotipo*			Excluído
	1° tercil	51,9	1	
	2° tercil	50,3	0,97 (0,78‐1,19)	
	3° tercil	57,7	1,14 (0,91‐1,36)	

*Sesta*				
	Nunca/quase nunca	44,8	1	1
	Às vezes	56,7	1,26 (1,04‐1,54)	1,13 (0,86‐1,49)
	Quase sempre/sempre	67,4	1,50 (1,16‐1,95)	1,23 (0,79‐1,92)

	*Atividade física*			Excluído
	Ativos	51,9	1	
	Insuficientemente ativos	56,2	1,08 (0,92‐1,27)	

*Tempo sentado*				
	1° tercil	47,3	1	1
	2° tercil	57,1	1,20 (1,00‐1,47)	1,01 (0,74‐1,38)
	3° tercil	61,2	1,29 (1,07‐1,57)	1,09 (0,80‐1,49)

PDSS, Pediatric Daytime Sleepiness Scale.

Outro fator fortemente associado à baixa duração de sono foi o turno de estudo. Os adolescentes que estudavam no turno da manhã apresentaram 1,82 (IC95% 1,35-2,46) vez maior prevalência de baixa duração de sono, comparados com os alunos do turno da tarde. Na análise ajustada, os adolescentes que estudavam no turno da noite apresentaram 1,68 (IC95% 1,14-2,46) vez mais prevalência de baixa duração do sono com relação aos adolescentes do turno da tarde.

A sonolência diurna, a sesta e o tempo sentado, embora não tenham permanecido associados com o desfecho na análise ajustada, apresentaram importantes associações com a baixa duração do sono. Os adolescentes do terceiro tercil da escala de sonolência apresentaram 1,26 (IC95% 1,03-1,56) vez mais prevalência de baixa duração de sono do que os do primeiro tercil. A sesta apresentou associação com a baixa duração de sono na categoria “às vezes” (RP=1,26; IC95% 1,04-1,54) e na categoria “sempre” (RP=1,50; IC95% 1,16-1,95). Isso indica que aqueles adolescentes com menor duração do sono noturno apresentaram mais frequência de sesta. Já o tempo sentado apresentou associações no segundo tercil (RP=1,20; IC95% 1,00-1,47) e no terceiro tercil (RP=1,29; IC95% 1,07-1,57). Isso indica que os adolescentes com maior tempo dedicado aos comportamentos sedentários tinham menor duração do sono durante a noite.

## Discussão

Este estudo apresentou evidências de associações da baixa duração do sono com o turno de estudo e a faixa etária dos adolescentes. A prevalência de baixa duração do sono foi de 53,6% entre os adolescentes estudados. Essa prevalência é elevada em comparação com a de outros estudos.^3^^,^ 9 ^,^ 12 ^,^ 30 Mesmo que a prevalência tenha sido alta, percentuais superiores foram observados em adolescentes americanos.^11^ Cabe ressaltar que o município de Maravilha (SC) é de pequeno porte e com grande percentual de população rural e tais prevalências não eram esperadas.

Os adolescentes que dormem pouco apresentam inúmeros riscos à saúde aumentados. Javaheri et al.^31^ alertam que a baixa duração do sono e a qualidade ruim do sono estão, em muitos casos, associadas à maior presença de patologias. Pereira et al.^10^ observaram relação entre a baixa duração de sono e a maior presença de estresse nos adolescentes. Além disso, conforme verificado no estudo de Nova et al.,^12^ adolescentes espanhóis com duração adequada de sono apresentaram menor incidência de alergias.

Observou-se tendência de aumento da prevalência de baixa duração do sono no decorrer da adolescência. Como descrito pela literatura, a idade está fortemente associada à baixa duração do sono, considerando o amadurecimento do sistema nervoso central e os comportamentos de risco, como o uso de mídias eletrônicas, especialmente à noite.^10^^,^ 32 Na adolescência, as pessoas estão mais propensas a apresentar um fenômeno biológico chamado atraso de fase do sono, no qual os adolescentes tendem a dormir e acordar mais tarde,^33^ o que pode colaborar para o aumento da sonolência diurna, caso o sono não seja recuperado durante o dia.^32^ Além disso, essa tendência biológica é exacerbada por determinados comportamentos que, somados aos compromissos escolares no início da manhã, aumentam de forma acentuada o número de adolescentes com baixa duração do sono.^4^

Pelas razões explicitadas, pode-se destacar que os adolescentes que frequentam os turnos de estudo da manhã e da noite apresentaram maiores associações com a baixa duração de sono. Adolescentes mais velhos e estudantes desses turnos constituem-se importante grupo de risco para a baixa duração do sono. No estudo de McKnight-Eily et al.,^11^ os autores propõem que, para amenizar as altas prevalências de baixa duração do sono, é necessária a observação individual dos adolescentes, para indicar a mudança de comportamentos e mesmo a troca de turno de estudo. Nesse contexto, Louzada e Menna-Barreto^34^ discutem a possibilidade de as aulas iniciarem em horários mais adiantados da manhã como possibilidade de intervenção. No entanto, os mesmo autores destacam a dificuldade de as escolas adotarem tais recomendações, considerando os horários de trabalho dos pais e mesmo a cultura de valorização das atividades que iniciam mais cedo.

Verificou-se que a maior frequência de sestas esteve associada com a baixa duração de sono nos adolescentes. Provavelmente os adolescentes com maior frequência de sesta usam esse hábito como forma de compensar a baixa duração do sono noturno. Esse resultado corrobora os achados de Bernardo et al.,^9^ que também observaram maior prevalência de baixa duração do sono naqueles adolescentes com maior frequência de sesta. Apesar de esse hábito ser uma forma de compensar débitos de sono, segundo Carskadon et al.,^2^ pode atrasar a propensão ao sono noturno, retardar o início e diminuir sua duração. Dessa forma, são necessárias investigações de dose-resposta da sesta, considerando sua frequência e duração para possíveis recomendações para adolescentes.

Destaca-se, por fim, que os adolescentes com menor duração do sono apresentaram maior tempo de comportamento sedentário. Assim como a literatura aponta para o aumento das prevalências de baixa duração do sono, também é possível identificar que os adolescentes passam cada vez mais tempo em comportamentos sedentários, especialmente em frente à TV e internet.^35^^,^ 36 Assim, é necessário investigar se aqueles adolescentes com menor duração do sono acabam preferindo ficar mais tempo em atividades com baixo dispêndio energético em função, por exemplo, da sonolência diurna excessiva.

Pontos positivos deste estudo podem ser destacados, como, por exemplo, o uso de amostra representativa de um município do interior de pequeno porte pouco estudado, bem como a discussão de associações ainda pouco disponíveis na literatura nacional. A principal limitação deste estudo é o uso de questionário para avaliar de forma indireta as questões de sono e atividade física que, apesar de ser instrumentos validados, podem levar a medidas subestimadas ou superestimadas pelos adolescentes.

Os adolescentes de Maravilha (SC) apresentaram alta prevalência de baixa duração do sono. Os mais velhos e aqueles que estudavam de manhã e à noite apresentaram duração do sono reduzida. Esse resultado remete à necessidade de ações voltadas para a saúde do escolar focadas nos perfis biológico e comportamental nas diferentes fases da adolescência. Nesse aspecto é importante destacar que o adolescente pode não perceber as alterações no seu sono e medidas educativas são importantes desde o início da educação básica, o que colabora para a criação e manutenção de adequada higiene do sono. Nesse sentido, recomenda-se que exista a oferta de turmas no período da tarde em todos os anos escolares e mesmo a criação de um turno intermediário com início no meio da manhã.

Mesmo que a idade e o turno tenham sido os fatores mais fortemente associados com baixa duração do sono, a sesta, os comportamentos sedentários e a sonolência diurna são importantes variáveis que devem ser consideradas em análises de baixa duração do sono. Assim, recomenda-se a realização de estudos que possibilitem o conhecimento das relações causais entre essas variáveis e que indiquem, com maior precisão, o tempo de sesta adequado para que o adolescente não tenha prejuízos do sono noturno. É fundamental também que se proponham formas de minimizar o efeito da privação do sono no aumento da sonolência diurna e do tempo dedicado aos comportamentos sedentários considerando a realidade na qual o adolescente está inserido.
